# Recommendations for Medical and Mental Health Care in Assisted Living Based on an Expert Delphi Consensus Panel: A Consensus Statement

**DOI:** 10.1001/jamanetworkopen.2022.33872

**Published:** 2022-09-01

**Authors:** Sheryl Zimmerman, Philip D. Sloane, Christopher J. Wretman, Kevin Cao, Johanna Silbersack, Paula Carder, Kali S. Thomas, Josh Allen, Kim Butrum, Tony Chicotel, Pat Giorgio, Mauro Hernandez, Helen Kales, Paul Katz, Juliet Holt Klinger, Margo Kunze, Christopher Laxton, Vicki McNealley, Suzanne Meeks, Kevin O’Neil, Douglas Pace, Barbara Resnick, Lindsay Schwartz, Dallas Seitz, Lori Smetanka, Kimberly Van Haitsma

**Affiliations:** Cecil G. Sheps Center for Health Services Research, University of North Carolina at Chapel Hill, Chapel Hill; School of Social Work, University of North Carolina at Chapel Hill, Chapel Hill; Gillings School of Global Public Health, University of North Carolina at Chapel Hill, Chapel Hill; Cecil G. Sheps Center for Health Services Research, University of North Carolina at Chapel Hill, Chapel Hill; Department of Family Medicine, School of Medicine, University of North Carolina at Chapel Hill, Chapel Hill; Wretman Research, LLC, Hillsborough; School of Medicine, University of Illinois Chicago, Chicago; Cecil G. Sheps Center for Health Services Research, University of North Carolina at Chapel Hill, Chapel Hill; Portland State University, Portland, Oregon; Brown University, Providence, Rhode Island; Allen Flores Consulting Group, Searcy, Arkansas; Silverado, Irvine, California; California Advocates for Nursing Home Reform, Berkeley; Evergreen Estates, Clarkston, Washington; Hearth & Truss, Wilsonville, Oregon; Department of Psychiatry, University of Michigan, Ann Arbor; Department of Geriatrics, College of Medicine, Florida State University, Tallahassee; Brookdale Senior Living, Brentwood, Tennessee; American Assisted Living Nurses Association, Belmar, New Jersey; Society for Post-Acute and Long-Term Care Medicine, Columbia, Maryland; Washington Health Care Association, Tumwater; Department of Psychological & Brain Sciences, University of Louisville, Louisville, Kentucky; ALG Senior, Hickory, North Carolina; Alzheimer’s Association, Chicago, Illinois; University of Maryland School of Nursing, Baltimore; Workforce & Quality Innovations, LLC, Bear Creek, North Carolina; Hotchkiss Brain Institute, University of Calgary, Calgary, Alberta, Canada; National Consumer Voice for Quality Long-Term Care, Washington, DC; College of Nursing, The Pennsylvania State University, University Park

## Abstract

**IMPORTANCE:**

Assisted living (AL) is the largest provider of residential long-term care in the US, and the morbidity of AL residents has been rising. However, AL is not a health care setting, and concern has been growing about residents’ medical and mental health needs. No guidance exists to inform this care.

**OBJECTIVE:**

To identify consensus recommendations for medical and mental health care in AL and determine whether they are pragmatic.

**EVIDENCE REVIEW:**

A Delphi consensus statement study was conducted in 2021; as a separate effort, the extent to which the recommendations are reflected in practice was examined in data obtained from 2016 to 2021 (prepandemic). In the separate effort, data were from a 7-state study (Arkansas, Louisiana, New Jersey, New York, Oklahoma, Pennsylvania, Texas). The 19 Delphi panelists constituted nationally recognized experts in medical, nursing, and mental health needs of and care for older adults; dementia care; and AL and long-term care management, advocacy, regulation, and education. One invitee was unavailable and nominated an alternate. The primary outcome was identification of recommended practices based on consensus ratings of importance. Panelists rated 183 items regarding importance to care quality and feasibility.

**FINDINGS:**

Consensus identified 43 recommendations in the areas of staff and staff training, nursing and related services, resident assessment and care planning, policies and practices, and medical and mental health clinicians and care. To determine the pragmatism of the recommendations, their prevalence was examined in the 7-state study and found that most were in practice. The items reflected the tenets of AL, the role of AL in providing dementia care, the need for pragmatism due to the diversity of AL, and workforce needs.

**CONCLUSIONS AND RELEVANCE:**

In this consensus statement, 43 recommendations important to medical and mental health care in AL were delineated that are highly pragmatic as a guide for practice and policy.

## Introduction

More than 1.6 million people in the US receive long-term care in nursing homes or assisted living (AL) communities; historically the majority resided in nursing homes, but now more than half of residential long-term care (not including postacute rehabilitation) is provided in AL.^1^ This shift reflects expansion responsive to consumer preference; the cost of nursing home care; restrictions on nursing home growth; the fact that older adults often require supportive but not skilled nursing care; and private-pay market incentives.^2,3^ AL provides room and board, at least 2 meals a day, around-the-clock supervision, and personal care; its intent is to promote person-centered (person-directed) care and quality of life through supportive and responsive services, choice, and a homelike environment.^1,4^ Despite common intent, there is great variability in AL: communities range in size from 4 to many hundreds of beds, offer varied services, and are state-regulated through 350 different licensure and/or policy approaches.^5,6^

Over time, forces have led to increasing medical condition acuity and care needs of AL residents. Hospital reimbursement based on diagnosis-related groups resulted in shorter hospital stays and more nursing home transfers, and an increasing proportion of nursing home residents requiring postacute care.^7,8^ In turn, residents who had been in nursing homes but whose conditions were manageable became the new face of AL. Today, 53% of AL residents are aged 85 years or older, compared with 42% in nursing homes.^9^ More than half need help with locomotion and have hypertension, arthritis, cognitive impairment, and depression, and one-third or more have osteoporosis, chronic obstructive pulmonary disease, diabetes, heart disease, and chronic kidney disease, and visit an emergency department each year.^1,10,11^ Their average length of stay is 22 months, and eventually 60% transition to a nursing home.^12^

As residents’ acuity has increased, there has been growing concern about their medical and mental health needs, in part because most regulations do not require nursing or medical staff; more so, fewer than 7% of the 28 900 AL communities are on the same campus as a nursing home, with presumed access to nursing home staff.^1,13^ Concerns are numerous, among them underprescribing or overprescribing medication, insufficient communication when problems arise, and regulations that restrict nursing services.^14–20^ In response, medical and mental health care in AL has been evolving; 54% of communities now have a registered nurse (RN) or licensed practical nurse (LPN) on staff, and some offer onsite medical care.^21,22^ Amidst this evolution, the optimal structures and processes of medical and mental health care in AL have not been determined—for example, the role of nurses and primary care practitioners, use of electronic medical records, and training for staff—and there is concern that if AL becomes medicalized, consequences may include an erosion of its intent, a call for federal oversight, increased cost, and reduced accessibility.^23–26^

To address this pressing issue, this study used a modified Delphi panel approach to develop consensus recommendations for the provision of medical and mental health care in AL. As a separate secondary effort to understand feasibility and pragmatism, it examined the extent to which the recommendations were reflected in actual practice.

## Methods

The Delphi technique is commonly used to develop consensus on best practice guidelines, recommendations, and quality indicators, including in geriatrics and long-term care.^27–35^ It develops consensus through iterative rounds of inquiry that build on previous rounds, each being anonymous to avoid social pressure and conformity to a dominant view; most often ratings are provided using a 9-point scale, with a common cut point for consensus being 75% agreement.^27,36^ This project was conducted in compliance with the Conducting and Reporting of Delphi Studies (CREDES) standards^27^ and the Standards for Quality Improvement Reporting Excellence (SQUIRE) reporting guideline.^37^

### Expert Panelists

Nineteen panelists were selected expressly to constitute a diverse group of nationally recognized experts in medical, nursing, and mental health needs of and care for older adults, dementia care, and AL and long-term care management, advocacy, regulation, and education. Half were known to the investigative team based on previous participation on advisory boards. All individuals received an email invitation; one was unavailable and nominated an alternate from her organization. Table 1 lists each panel member, their key expertise, affiliation, and a note regarding conflict of interest; panelists provided their race, sex, and age to more fully describe the participants.

### Process

Panelists participated in an initial videoconference discussion during which the study was overviewed. They then completed anonymous questionnaires via Qualtrics (2 rounds) and email (1 round), after which a final videoconference meeting was convened to discuss results. All panelists provided consent, and the study was approved by the University of North Carolina at Chapel Hill institutional review board.

#### Round 1:

Experts rated 183 items of potential importance to medical and mental health care in AL, grouped into 6 categories: (1) community demographics and administration, (2) staff and staff training, (3) nursing and related services, (4) resident assessment and care planning, (5) policies and practices, and (6) medical and mental health clinicians and care. The items were compiled from a comprehensive literature search and advisory panel for an ongoing 7-state study of medical and mental health care in AL, with additional items recommended during the initial videoconference meeting; none of the items were selected based on the extent to which they were evidenced in practice. More specifically, all items had either been included in previous AL or nursing home research as a potential covariate or outcome, and/or included in AL state regulations, and/or included in AL community guidelines, and/or considered of potential importance based on expert opinion (eTable 1 in the Supplement identifies each item accordingly). For the very reason that this study was conducted, the actual evidence base regarding important components of medical and mental health care is thin.

Panelists scored each item on 2 criteria: (1) Importance to quality of care: the extent to which the item is expected to substantially affect quality of care outcomes if implemented, considering the extent of expected need and magnitude of benefit, scored 1 to 9 (least to most important). (2) Feasibility: the extent to which the item is feasible for (can potentially be implemented in) no, some, or all AL communities, based on factors such as variable case mix, location, or other considerations, considered in the context of today’s AL environment.

#### Round 2:

Experts rated 3 items that required rewording and provided additional information for 17 items that were potentially unclear based on ratings from round 1 (eg, having a large standard deviation or unexpected scores). They also commented on recommendations that implicated a metric or cut point, such as ratios and percentages, providing a narrative response.

#### Round 3:

Experts rated 7 items that were further reworded.

### Analysis

The mean and standard deviation of all items were derived, and items were grouped into categories recommended by CREDES of high importance (scored at least 7.0), medium importance (at least 4.0 through less than 7.0), and low importance (less than 4.0); the percentage of respondents agreeing with each categorization was derived, with consensus set at greater than or equal to 75% as is common.^27,38^ In addition, because numerous items that were related to a like topic were scored similarly (eg, topics to include in staff training), aggregate categories for 5 topics were created from 22 separate items for the final tables.

To examine the extent to which the recommendations were actually feasible for practice and pragmatic, in a separate subsequent effort, data from a 7-state study of 250 AL communities were used to examine the prevalence of items considered important (scored at least 7.0) and achieving consensus (scored as such by at least 75% of panelists). The communities were representative of AL communities in the states,^39^ but because some items were available for only a subset of communities (n = 151), the prevalence findings are more illustrative than generalizable.

## Results

Among the 19 panelists, 18 (95%) were White, 8 (42%) identified as male, and the mean (SD) age was 59 (10) years. Work experience included providing clinical care to older adults and/or being an administrator of an AL community (74%), being affiliated with an AL community or other long-term care organization (69%), and being involved in AL regulation or oversight (53%).

The decision-making process is displayed in eTable 2 in the Supplement; R indicates the item was rated as important/recommended (scored at least 7.0 by at least 75% of panelists), C indicates it was rated important/worthy of consideration (scored at least 7.0 by less than 75% of panelists), L indicates it was rated of medium importance/limited utility (scored at least 4.0 through less than 7.0), and E indicates it was rated not important/excluded (scored less than 4.0).

Table 2 displays the 43 items constituting expert consensus recommendations for medical and mental health care in AL, organized based on importance and grouped into 5 categories: (1) staff and staff training, (2) nursing and related services, (3) resident assessment and care planning, (4) policies and practices, and (5) medical and mental health clinicians and care. (None of the items related to community demographics and administration met criteria for inclusion.) Five of the 43 items are aggregates and include footnotes detailing the individual items; the scores for those items are in eTable 3 in the Supplement.

The item most recommended (scored 8.89, 100% consensus) was in the domain of staff training: *training on person-centered care*. The highest recommendations in the other domains were *provision of routine toenail care* (scored 8.16, 89.5%), *resident present during assessment/care planning* (scored 8.32, 94.7%), *has a policy/procedure regarding aggressive or other behaviors* (scored 8.68, 100%), and *all off-site medical or mental health visits include post-visit notes with findings* (scored 8.59, 100%).

Three items related to staffing referred to an amount, but panelists did not recommend an exact amount. While noting the importance of the *direct care worker-to-resident ratio* (scored 8.68, 100% consensus), narrative responses indicated the ratio should be acuity-driven, related to care needs, and evidence-based as per its association with outcomes; panelists noted the need for research to determine the ratio. Similarly, the specific recommended *percent of direct care workers who are not contract staff*, and who are *full time*, could not be articulated, largely because panelists recognized the reality of staffing challenges and were loath to establish unrealistic recommendations despite the importance of a consistent workforce. Comments included “people split shifts so they can be home with their children” and “the cut point should be based on what is reasonable [as per] employment conditions.” Again, panelists noted the need for research to establish metrics based on outcomes.

Table 2 also indicates the perceived feasibility for each recommendation, with 1, 2, and 3 indicating feasible in no, some, or all AL communities. Roughly 75% of the items were rated 2.5 or higher, indicating it was considered feasible for between some and all AL communities (32 of 43 items). The lowest feasibility rating was 2.11 for *have an RN available on-site,* followed by *provision of occupational therapy on-site, have any medical care provided on-site,* and *have any mental health care provided on-site.*

Table 3 provides the same information for items rated of high importance but endorsed by fewer than 75% of panelists; many were endorsed by 74%, almost meeting the consensus cut point and therefore worthy of consideration. Of all items, the highest rated is *training for any staff on communicating with health care providers regarding change in status* (scored 7.68, 73.7% consensus, feasibility 2.74). eTables 4 and 5 in the Supplement provide information for the items considered of medium and low importance. In eTable 4 in the Supplement, the item *has a medical director or equivalent* scored 6.00 (medium importance) with a feasibility of 2.11; 68% of panelists agreed with that rating. Comments included potential benefits (eg, “overall medical leadership; ensure access to appropriate routine and emergency medical care; help lead infection control”) and also potential liabilities (eg, “cost would be passed onto the resident; residents should have choice to continue seeing their preferred physician; the mere presence of a medical director does not have a significant impact on quality”).

Given the recommendations in Table 2, it is helpful to understand the current availability of medical and mental health care so as to understand the extent to which they are feasible and pragmatic, and to serve as a benchmark going forward. In the separate 7-state study, data were available for 26 of the 43 items (61%) that met the thresholds of importance and consensus. Table 4 indicates that of those 26 items, 20 (77%) were evidenced in at least three-quarters of communities, including all items in the nursing and related services category; those that were least common were *having a program or policy for gradual dose reduction for psychotropic medications* (44%) and *conducting as needed formal care/service plan meetings* (11%). Of note, half of the communities reported at least biannual meetings.

## Discussion

To our knowledge, this study is the first to develop consensus recommendations for medical and mental health care in AL, a long-overdue effort given that AL is the largest provider of residential long-term care in the country, resident acuity has grown, and there have been calls to bolster medical and mental health care in AL for years.^1,23,24,26^ Those calls preceded the COVID-19 pandemic, which was largely responsible for an excess mortality rate of 17% and highlighted the importance of attending to the needs of AL residents and pointed out gaps in care.^40–42^

There was notable agreement among diverse experts on 43 recommendations for medical and mental health care in AL, regarding both importance and perceived feasibility. Feasibility is consequential not only because of the variability in AL, but also because many of the tensions inherent in AL—including resident acuity, regulatory complexity, cost and financing, workforce insufficiency, and models of AL—are at times in conflict.^26^ Thus, recommendations must be sensitive to the reality of AL. There is evidence the expert panelists were indeed sensitive to these realities, such as reflected in their mid-range rating regarding whether to recommend a medical director, which they explained as related to competing drivers of cost, choice, and quality.

A critical examination of the 43 consensus recommendations finds that in addition to detailing items within the 5 specified domains (ie, staff and staff training, nursing and related services, resident assessment and care planning, policies and practices, medical and mental health clinicians and care), 4 critical components of AL were embraced: the tenets of AL, the role of AL providing dementia care, the need for pragmatism in light of AL diversity, and workforce needs.

### Consensus Recommendations Reflect Tenets of AL

Given the intent of AL to promote person-centered care, quality of life, and aging-in-place,^2,4^ it is reassuring that experts agreed on the importance of staff training on person-centered care and end-of-life care and advance care planning; that care and service plan meetings be conducted as needed (not strictly on a prescribed schedule); that residents and direct care workers attend those meetings; that those meetings result in documented discussions about advance directives; and that able residents provide consent for new psychoactive medications.

### Consensus Recommendations Recognize AL as a Provider of Dementia Care

AL is the largest residential provider of long-term dementia care,^21^ and the recommendations reflect their needs. They address staff training for dementia; conducting cognitive assessments using a formal tool and when residents display agitation; having policies to manage behaviors, including a gradual dose reduction program for psychotropic medications; and involving a responsible party during assessment/care planning and informing that person when there is a change in status, medication, need for a new psychoactive medication, or emergency department visit.

### Consensus Recommendations Are Pragmatic and Respect the Diversity of AL

The pragmatic nature of the recommendations is evident in that of the 26 items for which data were available, 77% were practiced in at least three-quarters of communities, including all of the nursing and related services items. Clearly, data indicate that AL already provides some health care services, and at the same time indicate areas in which the field needs to progress. Two of the 4 items practiced by fewer than three-quarters of communities can be addressed without undue burden through operational management: staff training for dementia and mental illness, and conducting as needed formal assessments. The other 2 items implicate the need to increase the clinical workforce in AL: having an RN on site and a program or policy related to gradual dose reduction for psychotropic medications.

### Consensus Recommendations Identify Workforce Needs

Recommendations related to the workforce speak to having an RN or LPN on site and providing medical and mental health care on site. Nursing presence has become common, and more than half of communities have a nurse on staff.^21^ Physician and advanced practitioner presence has also become more common, but across the nation fewer than 12 000 clinicians provide care in AL.^43^ In addition, the need for mental health care clinicians is increasing, given that 11% of AL residents have serious mental illness (a 54% growth over a decade).^44^ Thus, there is need for more clinicians in AL, for both direct care and staff training. Also related to workforce is the need for data regarding direct care workers, including optimal staffing ratios based on resident acuity, and how best to use parttime and contract staff.

### Limitations

A key limitation of this work is that the recommendations relate to AL in the US, which differs from models in other countries; in addition, international models are themselves in flux. For example, residential long-term care in China is growing rapidly,^45^ whereas it is just emerging in Latin America,^46^ and in the Netherlands, recent reforms have challenged payment and accessibility.^47^ Therefore, although older adults across the globe have similar care needs,^48^ the recommendations do not necessarily have a parallel to models of residential care in other countries; nonetheless, a Delphi consensus panel akin to the one implemented in this study could help guide their efforts. A second limitation is that many of the recommendations require further specification, such as the content of training on person-centered care.

## Conclusions

The 43 consensus recommendations demonstrate notable agreement on components of medical and mental health care that are fine-tuned to AL. Other than tackling the need for a more sufficient workforce—which is relevant for all long-term care—the recommendations are highly pragmatic to guide practice and policy. Evidence is needed to determine the extent to which the recommendations improve outcomes, and also to which the components of care are feasible in a sample larger than that reflected in this paper. Feedback regarding medical and mental health care is needed from additional stakeholders as well, especially residents and families. As a notable strength of the panel, it included 19 individuals, and stability of response has been demonstrated in panels as small as 23 members.^49^

The recommendations are not currently being promoted as actual guidelines because the evidence base regarding benefits and harms is not yet developed; nonetheless, it is useful to situate the recommendations in the context of the Institute of Medicine’s standards for clinical practice guidelines. In this regard, the development of the recommendations is trustworthy in relation to being transparent, managing conflict of interest, and having purposeful group composition and clear articulation. Before actual guidelines can be promoted, it will be necessary to crosswalk the recommendations with the evidence and systematic review, solicit external review, and assure updating.^50^

The conclusions and implications are clear: implement the recommendations for medical and mental health care in AL, and in so doing, examine issues related to adoption (eg, needed resources) and outcomes (eg, fewer hospitalizations, less depression). As noted previously, implementation may require further specificity, and evaluation should be conducted in regard to the heterogeneity of AL. The recommendations must be conveyed to policy makers, those who own and manage AL, and professional, clinician, health care, and advocacy organizations—and also to prospective and current residents and family members, helping them become more informed when choosing a community and directing their care. The impetus for change may come from any of these stakeholders, and the more parties advocating for change, the more likely it is to occur.

When promoting the 43 recommendations, ratings on the remaining items also should be shared—meaning those items considered important but not by at least 75% of respondents (Table 3) and those rated of medium and low importance (eTables 4 and 5 in the Supplement). Some of those recommendations may be more important and feasible than currently rated for a specific type of AL community, such as in an urban area where resources are plentiful; or in communities that have a particular case-mix, such as residents receiving rehabilitation. Guidance has been long-awaited regarding medical and mental health care in AL; the consensus recommendations in this paper are an initial step to fill that gap.

## Supplementary Material

SUPPLEMENT eTable 1. Source of Items
eTable 2. Decision-making Process Across Delphi Rounds
eTable 3. Aggregate Items (included in Table 1) and Original Items (N=19)
eTable 4. Items Rated of Medium Importance (≥4.0, < 7.0), by Domain (N=19)
eTable 5. Items Rated of Low Importance (< 4.0), by Domain (N=19)

## Figures and Tables

**Table 1.** Panel Member Names, Expertise, Affiliation, and Note Regarding Potential Conflict of Interest^a^

Name	Key expertise	Affiliation
Josh Allen, RN	AL nursing	Allen Flores Consulting Group
Kim Butrum, RN, MS	AL management	Silverado
Tony Chicotel, JD, MPP	Long-term care advocacy	California Advocates for Nursing Home Reform
Pat Giorgio, MPS	AL management	Evergreen Estates
Mauro Hernandez, PhD	AL management	Hearth & Truss
Helen Kales, MD	Geriatric psychiatry	Department of Psychiatry, University of Michigan
Paul Katz, MD	Geriatrics	Department of Geriatrics, College of Medicine, Florida State University
Juliet Holt Klinger, MA	AL dementia care	Brookdale Senior Living
Margo Kunze, RN	AL nursing	American Assisted Living Nurses Association
Christopher Laxton, CAE	Long-term care medicine	Society for Post-Acute and Long-Term Care Medicine
Vicki McNealley, PhD, MN, RN	AL regulation	Washington Health Care Association
Suzanne Meeks, PhD	Mental health in late life	Department of Psychological & Brain Sciences, University of Louisville
Kevin O’Neil, MD	AL medical care	ALG Senior
Douglas Pace, NHA	Alzheimer disease/dementia care	Alzheimer’s Association
Barbara Resnick, PhD, RN	AL nursing	University of Maryland School of Nursing
Lindsay Schwartz, PhD	AL workforce and quality	Workforce & Quality Innovations
Dallas Seitz, MD, PhD	Geriatric psychiatry	Hotchkiss Brain Institute, University of Calgary
Lori Smetanka, JD	Advocacy	National Consumer Voice for Quality Long-Term Care
Kimberly Van Haitsma, PhD	Person-centered care	College of Nursing, The Pennsylvania State University

Abbreviation: AL, assisted living.

a The panelists were selected based on their involvement in the field, but none is in a position to benefit financially or personally based on their involvement in the panel or the resulting recommendations. Development of the recommendations explicitly excluded naming any products or organizations that might have constituted a conflict of interest for the panelists. Therefore, no conflicts of interest are reported.

**Table 2.** Expert Consensus Recommendations for Medical and Mental Health Care in Assisted Living: Items Rated of High Importance (≥7.0) by at Least 75% of the 19 Panelists, by Domain^a^

Domains and items	Importance, mean (SD)	% Agree importance ≥7.0	Feasibility, mean (SD)
Staffing and staff training			
Training for any staff on person-centered care	8.89 (0.32)	100.0	2.89 (0.32)
Direct care worker-to-resident ratio	8.68 (0.58)	100.0	2.84 (0.37)
Staff training for dementia/mental illness^b^	8.54 (0.55)	96.5	2.74 (0.42)
Training on side effects of drug treatments for staff who administer medications	8.53 (0.84)	94.7	2.89 (0.32)
Health care supervisor training and knowledge^c^	8.49 (0.78)	97.4	2.74 (0.45)
Training for any staff on infection prevention and control	8.42 (1.12)	94.7	2.95 (0.23)
% of direct care workers who are not contract staff	8.21 (1.23)	94.7	2.47 (0.51)
Training for any staff on end-of-life care/advance care planning	8.16 (1.12)	89.5	2.74 (0.45)
% of direct care workers who are full-time	7.95 (1.18)	84.2	2.68 (0.48)
Has RN available on-site	7.95 (1.54)	84.2	2.11 (0.32)
Has LPN/LVN available on-site	7.89 (1.20)	78.9	2.32 (0.48)
Nursing and related services			
Provision of routine toenail care on-site	8.16 (1.17)	89.5	2.58 (0.51)
Administration of influenza vaccines on-site	8.05 (1.54)	84.2	2.63 (0.50)
Provision of physical therapy on-site	7.94 (1.16)	88.9	2.26 (0.45)
Provision of insulin injections on-site	7.89 (2.13)	84.2	2.47 (0.51)
Blood glucose testing on-site	7.84 (1.38)	84.2	2.61 (0.50)
AL staff schedule residents’ medical and mental health care visits	7.74 (1.73)	78.9	2.53 (0.51)
Provision of occupational therapy on-site	7.73 (1.19)	84.2	2.16 (0.37)
Obtainment of weight for all residents at least monthly on-site	7.58 (2.48)	78.9	2.74 (0.56)
Administration of breathing/nebulizer treatments on-site	7.42 (1.92)	78.9	2.26 (0.56)
Resident assessment and care planning			
Resident present during assessment/care planning	8.32 (1.16)	94.7	2.74 (0.45)
Conducts a formal cognitive assessment as part of resident assessment	8.32 (1.11)	84.2	2.74 (0.45)
Nurse present during assessment/care planning	8.05 (1.31)	89.5	2.47 (0.51)
Uses a formal assessment tool for cognition	8.00 (1.53)	84.2	2.63 (0.50)
Conducts a standardized assessment to determine cause when a resident is agitated	8.00 (1.89)	89.5	2.53 (0.61)
Certified nursing assistant/personal care aide present during assessment/care planning	7.79 (2.04)	84.2	2.68 (0.48)
Uses other formal assessment tools (other than for cognition)^d^	7.63 (1.40)	81.1	2.74 (0.38)
Conducts as needed formal resident care or service plan meeting	7.63 (2.36)	78.9	2.95 (0.23)
Family present during assessment/care planning	7.58 (1.61)	78.9	2.53 (0.51)
Health care supervisor present during assessment/care planning	7.58 (1.77)	78.9	2.47 (0.51)
Policies and practices			
Has a policy/procedure regarding aggressive or other behaviors	8.68 (0.58)	100.0	2.79 (0.42)
Informs a responsible party when an emergency department visit occurs	8.67 (0.59)	100.0	2.88 (0.33)
Discussions about advance directives occur for all residents and are documented	8.65 (0.70)	100.0	2.94 (0.24)
Records health information in chart^e^	8.43 (0.74)	94.7	2.86 (0.29)
Has a policy/procedure regarding expression of suicidal thoughts	8.32 (1.06)	94.7	2.67 (0.49)
Informs a responsible party when change in status^f^	8.16 (1.20)	91.2	2.84 (0.34)
If resident cannot respond, family provides consent for new antipsychotic or opioid	8.00 (1.41)	88.2	2.88 (0.33)
Informs a responsible party when a medication is changed	7.74 (1.33)	84.2	2.67 (0.49)
If resident is able to respond, resident provides consent for new antipsychotic or opioid	7.65 (2.32)	82.4	2.82 (0.53)
Has a program or policy related to gradual dose reduction for psychotropic medications	7.21 (2.32)	78.9	2.50 (0.51)
Medical and mental health clinicians and care			
All off-site medical or mental health visits include post-visit notes with findings	8.59 (0.62)	100.0	2.82 (0.39)
Has any medical care provided on-site	7.84 (1.57)	89.5	2.22 (0.43)
Has any mental health care provided on-site	7.42 (1.89)	78.9	2.22 (0.43)

Abbreviations: AL, assisted living; LPN, licensed practical nurse; LVN, licensed vocational nurse; RN, registered nurse.

a The 43 items are ordered based on importance rating within domain, with ties sorted by standard deviation. A total of 37 of 43 items (86.0%) were rated by all 19 respondents; for other items, the number of respondents was 17 to 18. Importance reflects the extent to which the item is expected to substantially affect quality of care outcomes if implemented, considering the extent of expected need and the expected magnitude of benefit, scored 1 to 9, with 1 being least important and 9 being most important. Feasibility reflects the extent to which the item is feasible for (can potentially be implemented in) no AL communities, some AL communities, or all AL communities, based on factors such as variable case mix, location, or other considerations, scored as none (1), some (2), and all (3) communities.

b Aggregate of 3 items related to staff training on (1) caring for people with dementia, (2) caring for people with mental illness, and (3) nondrug practices to address agitation/behaviors; ratings are means of the original ratings.

c Aggregate of 4 items related to (1) training and (2) knowledge regarding nondrug treatments for behaviors and drug treatment side effects for behaviors; ratings are means of the original ratings.

d Aggregate of 5 items related to use of formal assessment tools for (1) depression, (2) pressure ulcer risk, (3) falls risk, (4) presence of advance directives, and (5) elopement risk; ratings are means of the original ratings.

e Aggregate of 7 items related to recording health information regarding (1) weight, (2) vital signs, (3) emergency department visits, (4) hospitalizations, (5) falls, (6) telephone contact with clinicians, and (7) behaviors of residents receiving antipsychotics; ratings are means of the original ratings.

f Aggregate of 3 items related to informing responsible parties of (1) changes to cognition/behavior/mood, (2) when a fall occurs, and (3) when there are other changes to medical status; ratings are means of the original ratings.

**Table 3.** Items Rated of High Importance (≥7.0) by <75% of 19 Panelists, by Domain

Domains and items^a^	Importance, mean (SD)	% Agree importance ≥7.0	Feasibility, mean (SD)
Staffing and staff training			
Training for any staff on communicating with health care providers re: change in status	7.68 (1.49)	73.7	2.74 (0.45)
If RN on-site, has RN available 24/7	7.37 (2.50)	73.7	2.11 (0.32)
Training for any staff on medication and medication side effects	7.05 (1.68)	63.2	2.68 (0.48)
If RN on-site, has RN on-call if on-site RN is not present	7.05 (2.01)	68.4	2.42 (0.51)
If RN on-site, has RN available full-time	7.05 (2.09)	63.2	2.11 (0.32)
If LPN/LVN on-site, has LPN/LVN available full-time	7.00 (1.76)	57.9	2.16 (0.37)
If LPN/LVN on-site, has LPN/LVN available 24/7	7.00 (2.26)	63.2	2.21 (0.42)
Nursing and related services			
Blood drawing done for blood tests on-site	7.21 (1.84)	68.4	2.16 (0.37)
Resident assessment and care planning			
Conducts as needed formal resident assessment	7.16 (2.77)	73.7	2.95 (0.23)
Policies and practices			
Health care clinician progress notes are available to AL staff	7.26 (2.18)	68.4	2.24 (0.44)
Pharmacist conducts formal medication review 4 or more times/y	7.21 (2.23)	73.7	2.29 (0.59)
Medical and mental health clinicians and care			
AL makes residents’ vital signs available during visits	7.53 (1.90)	73.7	2.44 (0.51)
Medical clinicians participate in quality improvement efforts	7.50 (1.92)	72.2	2.11 (0.32)
Limited number of consistent PCPs treat majority of residents who need medical care	7.05 (1.78)	68.4	2.26 (0.45)
AL staff accompanies clinicians during visits	7.00 (2.21)	73.7	2.11 (0.32)

Abbreviations: AL, assisted living; LPN, licensed practical nurse; LVN, licensed vocational nurse; PCP, primary care practitioner; RN, registered nurse.

a The 16 items are ordered based on importance rating within domain, with ties sorted by standard deviation. A total of 15 of 16 items (93.8%) were rated by all 19 respondents; for the other item, the number of respondents was 17. Importance reflects the extent to which the item is expected to substantially affect quality of care outcomes if implemented, considering the extent of expected need and the expected magnitude of benefit, scored 1 to 9, with 1 being least important and 9 being most important. Feasibility reflects the extent to which the item is feasible for (can potentially be implemented in) no AL communities, some AL communities, or all AL communities, based on factors such as variable case mix, location, or other considerations, scored as none (1), some (2), and all (3) communities.

**Table 4.** Proportion of Assisted Living Communities in 7-State Study Evidencing Items Rated of High Importance by at Least 75% of Panelists, by Domain^a^

Domains and items	No. (%)
Staffing and staff training	
Staff training for dementia/mental illness^b^	92 (60.5)
Training on side effects of drug treatments for staff who administer medications	202 (81.5)
Health care supervisor training and knowledge^c^	191 (76.4)
Has RN available on-site^d^	152 (61.0)
Has LPN/LVN available on-site^d^	205 (82.3)
Nursing and related services	
Provision of routine toenail care on-site	146 (96.1)
Administration of influenza vaccines on-site	137 (90.1)
Provision of physical therapy on-site	151 (99.3)
Provision of insulin injections on-site	130 (85.5)
Blood glucose testing on-site	131 (86.2)
AL staff schedule residents’ medical and mental health care visits	134 (88.2)
Provision of occupational therapy on-site	151 (99.3)
Administration of breathing/nebulizer treatments on-site	135 (88.8)
Resident assessment and care planning	
Conducts a formal cognitive assessment as part of resident assessment	188 (75.2)
Uses a formal assessment tool for cognition	132 (86.8)
Conducts a standardized assessment to determine cause when a resident is agitated	172 (68.8)
Other formal assessment tools are used (other than for cognition)^e^	149 (98.0)
Conducts as needed formal resident care or service plan meeting	16 (10.5)
Policies and practices	
Has a policy/procedure regarding aggressive or other behaviors	143 (94.7)
Records health information in chart^f^	152 (100.0)
Has a policy/procedure regarding expression of suicidal thoughts	140 (92.1)
Informs a responsible party when change in status^g^	150 (98.7)
Informs a responsible party when a medication is changed	114 (75.0)
Has a program or policy related to gradual dose reduction for psychotropic medications	110 (44.2)
Medical and mental health clinicians and care	
Has any medical care provided on-site	126 (82.9)
Has any mental health care provided on-site	145 (66.2)

Abbreviations: AL, assisted living; LPN, licensed practical nurse; LVN, licensed vocational nurse; RN, registered nurse.

a Data are derived from a 7-state study of 250 assisted living communities; sample sizes range from 151–250.

b Defined herein as training for both dementia and mental illness.

c Defined herein as any training and having “some” knowledge of both nondrug treatments for behavioral symptoms and drug treatment side effects.

d Defined herein as being in nondementia area and/or dementia area.

e Defined herein as using any assessment tool for (1) depression, (2) pressure ulcer risk, (3) falls risk, or (4) presence of advance directives.

f Defined herein as recording of information related to (1) vital signs, (2) emergency department visits, (3) hospitalizations, (4) falls, (5) telephone contacts, or (6) behaviors for people receiving antipsychotics.

g Defined herein as specifically medical status.

## Key Points

### Question

What medical and mental health care should be provided in assisted living?

### Findings

This Delphi consensus statement study involved 19 experts who rated 183 items of potential importance to medical and mental health care in assisted living. Consensus identified 43 recommendations related to staff and staff training, nursing and related services, resident assessment and care planning, policies and practices, and medical and mental health clinicians and care.

### Meaning

The 43 recommendations provide a pragmatic guide for practice and policy regarding medical and mental health care in assisted living.
