# Supplementary material for: Recommendations for Medical and Mental Health Care in Assisted Living Based on an Expert Delphi Consensus Panel: A Consensus Statement
Source: JAMA Netw Open. Author manuscript; Available in PMC 2023 Jul 17. (PMC10350914; doi:10.1001/jamanetworkopen.2022.33872)
Supplement: SUPPLEMENT — eTable 1. Source of Items eTable 2. Decision-making Process Across Delphi Rounds eTable 3. Aggregate Items (included in Table 1) and Original Items (N=19) eTable 4. Items Rated of Medium Importance (≥4.0, < 7.0), by Domain (N=19) eTable 5. Items Rated of Low Importance (< 4.0), by Domain (N=19) [file NIHMS1893021-supplement-SUPPLEMENT.pdf]

Supplemental Online Content

Zimmerman S, Sloane PD, Wretman CJ, et al. Recommendations for medical and mental health care in assisted living based on an expert Delphi consensus panel: a consensus statement. *JAMA Netw Open.* 2022;5(9):e2233872. doi:10.1001/jamanetworkopen.2022.33872

eTable 1. Source of Items

eTable 2. Decision-making Process Across Delphi Rounds

eTable 3. Aggregate Items (included in Table 1) and Original Items (N=19)

eTable 4. Items Rated of Medium Importance (≥ 4.0, < 7.0), by Domain (N=19)

eTable 5. Items Rated of Low Importance (< 4.0), by Domain (N=19)

This supplemental material has been provided by the authors to give readers additional information about their work.

© 2022 Zimmerman S et al. *JAMA Network Open.*

eTable 1. Source of Items

Domains and Items	Literature	Assisted Living Regulation	Assisted Living Guideline	Expert Opinion
**Staffing & Staff Training**				
**Registered nurse (RN) staffing**				
Has RN available on-site	X	X	X	X
If RN on-site, has RN available 24/7	X	X		
If RN on-site, has RN available full-time	X	X		
If RN on-site, has RN available part-time	X	X		
If RN on-site, has RN on-call if on-site RN is not present	X	X	X	X
**Licensed practical nurse/licensed vocational nurse (LPN/LVN) staffing**				
Has LPN/LVN available on-site	X	X	X	
If LPN/LVN on-site, has LPN/LVN available 24/7	X	X		
If LPN/LVN on-site, has LPN/LVN available full-time	X	X		
If LPN/LVN on-site, LPN/LVN available part-time	X	X		
If LPN/LVN on-site, LPN/LVN on-call (if on-site LPN/LVN is not present)	X	X		
**Direct care worker (nursing assistant/personal care aide) staffing**				
Percent of direct care workers who are full-time	X	X		X
Percent of direct care workers who are contract staff	X	X		X
Percent of direct care workers who are NOT contract staff	X	X		X
Percent of direct care workers who are medication technicians		X		
Direct care worker-to-resident ratio	X	X	X	X
**Training**				
Health care supervisor training and knowledge	X	X		X
Has had training about non-drug treatments for behaviors	X	X	X	X
Extent of knowledge regarding non-drug treatments for behaviors	X	X	X	X
Has had training about the side effects of drug treatment for behaviors	X	X		
Extent of knowledge regarding drug treatment side effects for behaviors	X	X		X
Training on side effects of drug treatments for staff who administer medications	X	X	X	X
Training on side effects of drug treatment for staff who do not administer medications	X			X
Staff training for dementia/mental illness	X	X	X	X
Training for any staff on caring for people with dementia	X	X	X	X
Training for any staff on caring for people with mental illness	X	X		X
Training for any staff on non-drug practices to address agitation/behaviors	X	X	X	X
Training for any staff on person-centered care	X	X	X	X
Training for any staff on end-of-life care/advance care planning	X	X	X	X
Training for any staff on infection prevention and control	X	X	X	X
Training for any staff on communicating with health care providers re: change in status	X	X	X	X
Training for any staff on medication and medication side effects	X	X	X	X
**Nursing & Related Services**				
**On-site services**				
Provision of routine toenail care on-site	X			
Administration of influenza vaccines on-site	X		X	X
Provision of physical therapy on-site	X			X
Provision of insulin injections on-site	X	X		X
Blood sugar testing on-site	X	X		
Provision of occupational therapy on-site	X			
Obtainment of weight for all residents at least monthly on-site	X			X
Administration of breathing/nebulizer treatments on-site	X	X		X
Blood drawing done for blood tests on-site	X			X
Mobile x-ray comes on-site	X			X
Catheter insertion to obtain urine specimen on-site	X			
Provision of intramuscular injections on-site	X	X		
Administration of intravenous fluids on-site	X			

© 2022 Zimmerman S et al. *JAMA Network Open.*

Domains and Items	Literature	Assisted Living Regulation	Assisted Living Guideline	Expert Opinion
Administration of tube feedings on-site	X	X		
Provision of care for stage 2/3 pressure ulcers on-site	X	X		
**Assisted living (AL) staff support**				
AL staff schedule residents' medical and mental health care visits	X			X
If schedule off-site visits, AL provides transportation for off-site visits	X		X	
If schedule off-site visits, AL staff accompany residents for off-site visits	X		X	
**Resident Assessment & Care Planning**				
**Assessment**				
Conducts a formal cognitive assessment as part of resident assessment	X	X	X	X
Conducts a standardized assessment to determine cause when a resident is agitated	X	X		X
Conducts as needed formal resident assessment	X	X	X	X
Conducts quarterly formal resident assessment	X	X		X
Conducts twice a year formal resident assessment	X	X		X
Conducts annual formal resident assessment	X	X	X	X
**Assessment tools**				
Uses a formal assessment tool for cognition	X	X	X	X
Uses other formal assessment tools (for other than for cognition)	X		X	X
Uses formal assessment tool for depression	X		X	X
Uses formal assessment tool for pressure ulcer risk	X		X	X
Uses formal assessment tool for falls risk	X		X	X
Uses formal assessment tool for presence of advance directives	X		X	X
Uses formal assessment tool for elopement risk			X	X
**Care/service planning**				
Conducts as needed formal resident care or service plan meeting	X	X	X	X
Conducts quarterly formal resident care or service plan meetings	X		X	X
Conducts twice a year formal resident care or service plan meetings	X		X	X
Conducts annual formal resident care or service plan meetings	X	X	X	X
**Involvement**				
Resident present during assessment/care planning	X	X	X	X
Family present during assessment/care planning	X	X	X	X
Nurse present during assessment/care planning	X	X		X
Health care supervisor present during assessment/care planning	X	X	X	X
Certified nursing assistant/personal care aide present during assessment/care planning		X	X	X
Primary care provider (if medical needs) present during assessment/care planning				X
Mental health provider (if related needs) present during assessment/care planning				X
Rehabilitation provider (if related needs) present during assessment/care planning				X
**Policies & Practices**				
**General**				
Policy/procedure regarding aggressive or other behaviors	X	X	X	X
Policy/procedure regarding expression of suicidal thoughts		X	X	X
Program/policy related to gradual dose reduction for psychotropic medications	X		X	X
Policy/procedure regarding marijuana use	X	X	X	X
Policy/procedure requiring residents to have PRN (as needed) medication for agitation	X	X	X	X
Policy/procedure requiring residents to have PRN medication for insomnia	X		X	X
Antibiotic stewardship program in place	X		X	X
Requires all residents to have advance directives	X	X	X	X
Discussions about advance directives occur for all residents and are documented	X	X	X	X
Policy/procedure requiring a visit to an emergency department after a fall	X		X	X
Standard policies are only for residents who are not cognitively intact	X			
**Medication review**				
Pharmacist never conducts formal medication review	X	X		
Pharmacist conducts formal medication review 1-3 times a year	X	X	X	X
Pharmacist conducts formal medication review four or more times/year	X	X	X	X
Pharmacist conducts formal medication review = 12+ times a year	X			
**Charting**				
Records health information in chart	X	X	X	X
Weight recorded in chart		X	X	X
Vital signs recorded in chart	X	X	X	X

© 2022 Zimmerman S et al. *JAMA Network Open.*

Domains and Items	Literature	Assisted Living Regulation	Assisted Living Guideline	Expert Opinion
Emergency department visits recorded in chart	X	X	X	X
Hospitalizations recorded in chart	X	X	X	X
Falls recorded in chart	X	X	X	X
Telephone contact with medical providers recorded in chart	X		X	X
Behavior of residents receiving antipsychotics recorded in chart	X		X	X
Food consumption recorded in chart			X	X
Health care provider progress notes are available to AL staff	X			X
RN most commonly documents residents' care	X	X		
LPN/LVN most commonly documents residents' care	X	X		
Medication technician most commonly documents residents' care	X	X		
Certified nursing assistant most commonly documents residents' care	X	X	X	X
Personal care aide most commonly documents residents' care	X	X	X	X
**Communication**				
Informs a responsible party when change in status	X	X	X	X
Informs a responsible party when there are changes to cognition, behavior, or mood	X	X	X	X
Informs a responsible party when a fall occurs	X	X	X	X
Informs a responsible party when there are other changes to medical status	X	X	X	X
Informs a responsible party when a medication is changed	X	X	X	X
Informs a responsible party when an emergency department visit occurs	X	X	X	X
Informs a responsible party when on-site health care provider visits	X			X
**Consent**				
Requires informed consent from residents before prescribing a new medication		X	X	X
Requires informed consent from residents for new antipsychotic medications		X	X	X
Requires informed consent from residents for new opioid medications				X
If resident is able to respond, resident provides consent for new antipsychotic or opioid		X		X
Requires informed consent from families before prescribing a new medication	X		X	X
Requires informed consent from families for new antipsychotic medications	X		X	X
Requires informed consent from families for new opioid medications	X			X
If resident cannot respond, family provides consent for new antipsychotic or opioid			X	X
**Electronic records**				
Uses electronic health records for medication prescribing and administration	X		X	X
Uses electronic health records for resident care notes written by AL staff	X		X	X
Uses electronic health records for resident care notes written by health care provider	X			X
**Medical and Mental Health Providers & Care**				
**On-site care**				
Has any medical care provided on-site	X	X	X	X
If medical care is provided on-site, it is provided at least once/week	X	X	X	X
Percent of residents receiving on-site care	X		X	X
Percent of primary care providers (PCPs) who come on-site	X		X	X
PCP is on-site at least weekly	X			X
PCP is on-site at least monthly	X		X	X
Has any mental health care provided on-site	X	X	X	X
If mental health care is on-site, it is provided at least once/week	X			
Percent of residents needing mental health care who receive on-site care	X			X
Percent of mental health providers who come on-site	X			X
A mental health provider is on-site at least weekly	X			
A mental health provider is on-site at least monthly	X		X	X
AL makes residents' vital signs available during visits	X			X
AL staff accompanies providers during visits	X			X
Has a special room dedicated for care	X		X	X
**Off-site care**				
All off-site medical or mental health visits include post-visit notes with findings	X			X
**Providers**				
Limited number of consistent PCPs treat majority of residents who need medical care	X		X	X
Residents receive care from same PCP as prior to admit	X	X	X	X
Resident-to-unique PCP ratio	X			
Has PCPs who treat only long-term care residents	X			

© 2022 Zimmerman S et al. *JAMA Network Open.*

Domains and Items	Literature	Assisted Living Regulation	Assisted Living Guideline	Expert Opinion
PCP works in a practice that treats only long- term care residents	X			X
Medical doctor (MD)/doctor of osteopathy (DO) treats majority of residents	X		X	X
Nurse practitioner (NP) treats majority of residents	X			X
Physician assistant (PA) treats majority of residents	X			X
Has a medical director or equivalent	X		X	X
If medical director, is an MD	X		X	X
If medical director, is on-site at least weekly	X		X	X
If medical director, is on-site at least monthly	X		X	X
If medical director, practice only serves long-term care residents	X			X
Limited number of consistent mental health providers treat majority of residents who need mental health care	X			
Residents receive mental health care from same provider as prior to admit	X			X
Residents requiring mental health care-to-unique mental health provider ratio	X			
Has mental health providers who treat only long-term care residents	X			X
A mental health provider works in a practice that treats only long-term care residents	X			X
Psychiatrist treats majority of residents	X			X
Psychologist treats majority of residents	X			X
Social worker treats majority of residents	X			X
Counselor treats majority of residents	X			X
**Involvement**				
Medical providers participate in quality improvement efforts	X			X
Medical providers provide in-service training to staff	X			X
Mental health providers participate in quality improvement efforts	X			X
Mental health providers provide in-service training to staff	X			X
**Communication**				
Uses telemedicine with medical/mental health providers	X		X	X
Uses fax to communicate with medical/mental health providers	X		X	X
Uses text message to communicate with medical/mental health providers	X			X
Uses e-mail to communicate with medical/mental health providers	X		X	X
**Community Demographics & Administration**				
**AL community**				
AL community is affiliated with a nursing home	X			
AL community is affiliated with a hospital	X			
AL or parent organization operates a home care agency	X		X	
AL community has memory care unit/designated dementia beds	X	X		X
**Administrator**				
Number of years as administrator at that AL community	X			X
Administrator is a licensed nursing home administrator	X	X		
Administrator is a LPN/LVN	X			X
Administrator is a RN	X	X		X
Administrator has a Bachelor of Science in Nursing	X		X	X
Administrator has a Master of Science in Nursing	X			
Administrator is an Advanced Practice Nurse	X	X		
Administrator is a Physician Assistant	X			
**Health care supervisor**				
Number of years as health care supervisor at that AL community	X			X
Health care supervisor is a RN	X	X	X	X
Health care supervisor is a LPN/LVN	X	X	X	X
Health care supervisor has a Bachelor of Science in Nursing	X			X
Health care supervisor has a Master of Science in Nursing	X			
Health care supervisor is an Advanced Practice Nurse	X			X
Health care supervisor is a Physician Assistant	X			X
Health care supervisor is a licensed nursing home administrator	X	X		

Notes: A check mark indicates that the item has been noted in literature, regulation, guideline, and/or expert opinion. Because a Comprehensive review of all sources has not been conducted, the *absence* of a check mark does not indicate that the item has *not* been noted in literature, regulation, guideline, and/or expert opinion.

- Literature = included in one or more assisted living or nursing home research studies as a potential covariate or outcome
- Regulation = included in one or more assisted living state regulations

© 2022 Zimmerman S et al. *JAMA Network Open.*

- Guideline= included in one or more assisted living community guidelines
- Expert opinion = considered of potential importance based on expert opinion

© 2022 Zimmerman S et al. *JAMA Network Open.*

eTable 2. Decision-making Process Across Delphi Rounds

Domains and Items	Round 1	Round 2	Round 3	Notes
**Staffing & Staff Training (N=29 items)**				
**Registered nurse (RN) staffing**				
Has RN available on-site	R			
If RN on-site, has RN available 24/7	C			
If RN on-site, has RN available full-time	C			
If RN on-site, has RN available part-time	L			
If RN on-site, has RN on-call if on-site RN is not present	C			
**Licensed practical nurse/licensed vocational nurse (LPN/LVN) staffing**				
Has LPN/LVN available on-site	R			
If LPN/LVN on-site, has LPN/LVN available 24/7	C			
If LPN/LVN on-site, has LPN/LVN available full-time	C			
If LPN/LVN on-site, LPN/LVN available part-time	L			
If LPN/LVN on-site, LPN/LVN on-call (if on-site LPN/LVN is not present)	L			
**Direct care worker (nursing assistant/personal care aide) staffing**				
Percent of direct care workers who are full-time	R			
Percent of direct care workers who are contract staff	L	L		
Percent of direct care workers who are NOT contract staff		R		
Percent of direct care workers who are medication technicians	L	L		
Direct care worker-to-resident ratio	R			
**Training**				
Health care supervisor training and knowledge				Aggregate
Has had training about non-drug treatments for behaviors	R			
Extent of knowledge regarding non-drug treatments for behaviors	R			
Has had training about the side effects of drug treatment for behaviors	R			
Extent of knowledge regarding drug treatment side effects for behaviors	R			
Training on side effects of drug treatments for staff who administer medications	R			
Training on side effects of drug treatment for staff who do not administer medications	L			
Staff training for dementia/mental illness				Aggregate
Training for any staff on caring for people with dementia	R			
Training for any staff on caring for people with mental illness	R			
Training for any staff on non-drug practices to address agitation/behaviors	R			
Training for any staff on person-centered care	R			
Training for any staff on end-of-life care/advance care planning	R			
Training for any staff on infection prevention and control	R			
Training for any staff on communicating with health care providers re: change in status	C			
Training for any staff on medication and medication side effects	C			
**Nursing & Related Services (N=18 items)**				
**On-site services**				
Provision of routine toenail care on-site	R			
Administration of influenza vaccines on-site	R			
Provision of physical therapy on-site	R			
Provision of insulin injections on-site	R			
Blood sugar testing on-site	R			
Provision of occupational therapy on-site	R			
Obtainment of weight for all residents at least monthly on-site	R			
Administration of breathing/nebulizer treatments on-site	R			
Blood drawing done for blood tests on-site	C			
Mobile x-ray comes on-site	L			
Catheter insertion to obtain urine specimen on-site	L			
Provision of intramuscular injections on-site	L			
Administration of intravenous fluids on-site	L			
Administration of tube feedings on-site	L			
Provision of care for stage 2/3 pressure ulcers on-site	L			
**Assisted living (AL) staff support**				
AL staff schedule residents' medical and mental health care visits	R			
If schedule off-site visits, AL provides transportation for off-site visits	L			
If schedule off-site visits, AL staff accompany residents for off-site visits	L			

© 2022 Zimmerman S et al. *JAMA Network Open.*

Domains and Items	Round 1	Round 2	Round 3	Notes
**Resident Assessment & Care Planning (N=24 items)**				
**Assessment**				
Conducts a formal cognitive assessment as part of resident assessment	R			
Conducts a standardized assessment to determine cause when a resident is agitated	R			
Conducts as needed formal resident assessment	C			
Conducts quarterly formal resident assessment	L			
Conducts twice a year formal resident assessment	L			
Conducts annual formal resident assessment	L			
**Assessment tools**				
Uses a formal assessment tool for cognition	R			
Uses other formal assessment tools (for other than for cognition)				Aggregate
Uses formal assessment tool for depression	R			
Uses formal assessment tool for pressure ulcer risk	R			
Uses formal assessment tool for falls risk	R			
Uses formal assessment tool for presence of advance directives	R			
Uses formal assessment tool for elopement risk	C			
**Care/service planning**				
Conducts as needed formal resident care or service plan meeting	R			
Conducts quarterly formal resident care or service plan meetings	L	L	L	
Conducts twice a year formal resident care or service plan meetings	L	L		
Conducts annual formal resident care or service plan meetings	L	L		
**Involvement**				
Resident present during assessment/care planning	R			
Family present during assessment/care planning	R			
Nurse present during assessment/care planning	R			
Health care supervisor present during assessment/care planning	R			
Certified nursing assistant/personal care aide present during assessment/care planning	R			
Primary care provider (if medical needs) present during assessment/care planning	L			
Mental health provider (if related needs) present during assessment/care planning	L			
Rehabilitation provider (if related needs) present during assessment/care planning	L			
**Policies & Practices (N=46 items)**				
**General**				
Policy/procedure regarding aggressive or other behaviors	R			
Policy/procedure regarding expression of suicidal thoughts	R			
Program/policy related to gradual dose reduction for psychotropic medications	R			
Policy/procedure regarding marijuana use	L			
Policy/procedure requiring residents to have PRN (as needed) medication for agitation	L	L		
Policy/procedure requiring residents to have PRN medication for insomnia	L	L		
Antibiotic stewardship program in place	L			
Requires all residents to have advance directives	L			
Discussions about advance directives occur for all residents and are documented			R	
Policy/procedure requiring a visit to an emergency department after a fall	E			
Standard policies are only for residents who are not cognitively intact	E			
**Medication review**				
Pharmacist never conducts formal medication review	E			
Pharmacist conducts formal medication review 1-3 times a year	L			
Pharmacist conducts formal medication review four or more times/year	C			
Pharmacist conducts formal medication review = 12+ times a year	L			
**Charting**				
Records health information in chart				Aggregate
Weight recorded in chart	R			
Vital signs recorded in chart	R			
Emergency department visits recorded in chart	R			
Hospitalizations recorded in chart	R			
Falls recorded in chart	R			
Telephone contact with medical providers recorded in chart	R			
Behavior of residents receiving antipsychotics recorded in chart	**R**			
Food consumption recorded in chart	L			
Health care provider progress notes are available to AL staff	C			

© 2022 Zimmerman S et al. *JAMA Network Open.*

Domains and Items	Round 1	Round 2	Round 3	Notes
RN most commonly documents residents' care	L			
LPN/LVN most commonly documents residents' care	L			
Medication technician most commonly documents residents' care	L			
Certified nursing assistant most commonly documents residents' care	L			
Personal care aide most commonly documents residents' care	L			
**Communication**				
Informs a responsible party when change in status				Aggregate
Informs a responsible party when there are changes to cognition, behavior, or mood	R			
Informs a responsible party when a fall occurs	R			
Informs a responsible party when there are other changes to medical status	R			
Informs a responsible party when a medication is changed	R			
Informs a responsible party when an emergency department visit occurs	R			
Informs a responsible party when on-site health care provider visits	L			
**Consent**				
Requires informed consent from residents before prescribing a new medication	L			
Requires informed consent from residents for new antipsychotic medications	L	L		
Requires informed consent from residents for new opioid medications	L	L		
If resident is able to respond, resident provides consent for new antipsychotic or opioid			R	
Requires informed consent from families before prescribing a new medication	L			
Requires informed consent from families for new antipsychotic medications	L	L		
Requires informed consent from families for new opioid medications	L	L		
If resident cannot respond, family provides consent for new antipsychotic or opioid			R	
**Electronic records**				
Uses electronic health records for medication prescribing and administration	L			
Uses electronic health records for resident care notes written by AL staff	L			
Uses electronic health records for resident care notes written by health care provider	L			
**Medical and Mental Health Providers & Care (N=46 items)**				
**On-site care**				
Has any medical care provided on-site	R			
If medical care is provided on-site, it is provided at least once/week			L	
Percent of residents receiving on-site care	L	L		
Percent of primary care providers (PCPs) who come on-site	L	L		
PCP is on-site at least weekly	L	L		
PCP is on-site at least monthly	L	L		
Has any mental health care provided on-site	R			
If mental health care is on-site, it is provided at least once/week	L		L	
Percent of residents needing mental health care who receive on-site care	L	L		
Percent of mental health providers who come on-site	L	L		
A mental health provider is on-site at least weekly	L	L		
A mental health provider is on-site at least monthly	L	L		
AL makes residents' vital signs available during visits	C			
AL staff accompanies providers during visits	C			
Has a special room dedicated for care	L			
**Off-site care**				
All off-site medical or mental health visits include post-visit notes with findings			R	
**Providers**				
Limited number of consistent PCPs treat majority of residents who need medical care		C		
Residents receive care from same PCP as prior to admit	L			
Resident-to-unique PCP ratio	L	L		
Has PCPs who treat only long-term care residents	L			
PCP works in a practice that treats only long- term care residents	L			
Medical doctor (MD)/doctor of osteopathy (DO) treats majority of residents	L			
Nurse practitioner (NP) treats majority of residents	L			
Physician assistant (PA) treats majority of residents	L			
Has a medical director or equivalent	L	L		
If medical director, is an MD	L			
If medical director, is on-site at least weekly	L			
If medical director, is on-site at least monthly	L			
If medical director, practice only serves long-term care residents	L			

© 2022 Zimmerman S et al. *JAMA Network Open.*

Domains and Items	Round 1	Round 2	Round 3	Notes
Limited number of consistent mental health providers treat majority of residents who need mental health care		L		
Residents receive mental health care from same provider as prior to admit	L			
Residents requiring mental health care-to-unique mental health provider ratio	L	L		
Has mental health providers who treat only long-term care residents	L			
A mental health provider works in a practice that treats only long-term care residents	L			
Psychiatrist treats majority of residents	L			
Psychologist treats majority of residents	L			
Social worker treats majority of residents	L			
Counselor treats majority of residents	L			
**Involvement**				
Medical providers participate in quality improvement efforts	C			
Medical providers provide in-service training to staff	L			
Mental health providers participate in quality improvement efforts	L			
Mental health providers provide in-service training to staff	L			
**Communication**				
Uses telemedicine with medical/mental health providers	L			
Uses fax to communicate with medical/mental health providers	L			
Uses text message to communicate with medical/mental health providers	L			
Uses e-mail to communicate with medical/mental health providers	L			
**Community Demographics & Administration (N=20 items)**				
**AL community**				
AL community is affiliated with a nursing home	E			
AL community is affiliated with a hospital	E			
AL or parent organization operates a home care agency	E			
AL community has memory care unit/designated dementia beds	L			
**Administrator**				
Number of years as administrator at that AL community	L			
Administrator is a licensed nursing home administrator	L			
Administrator is a LPN/LVN	L			
Administrator is a RN	L			
Administrator has a Bachelor of Science in Nursing	L			
Administrator has a Master of Science in Nursing	L			
Administrator is an Advanced Practice Nurse	L			
Administrator is a Physician Assistant	L			
**Health care supervisor**				
Number of years as health care supervisor at that AL community	L			
Health care supervisor is a RN	L			
Health care supervisor is a LPN/LVN	L			
Health care supervisor has a Bachelor of Science in Nursing	L			
Health care supervisor has a Master of Science in Nursing	L			
Health care supervisor is an Advanced Practice Nurse	L			
Health care supervisor is a Physician Assistant	L			
Health care supervisor is a licensed nursing home administrator	E			

Note: R = Recommend; rated as high importance (≥ 7.0) by ≥ 75% of panelists
C = Consider; rated of high importance (≥ 7.0) by <75% of panelists
L = Limited; rated of medium importance (≥ 4.0, < 7.0) by panelists
E = Exclude; rated of low importance (< 4.0) by panelists
Aggregate is an item that was developed after Round 3 to include the sub-items listed

© 2022 Zimmerman S et al. *JAMA Network Open.*

eTable 3. Aggregate Items (included in Table 1) and Original Items (N=19)

Items	Importance 1–9 Mean (SD)	Percent Agreeing with Rating ≥7.0	Feasibility 1–3 Mean (SD)
**Staff training for dementia/mental illness**	**8.54 (0.55)**	**96.5**	**2.74 (0.42)**
Training for any staff on caring for people with dementia	8.74 (0.56)	100.0	2.84 (0.37)
Training for any staff on caring for people with mental illness	8.21 (1.08)	94.7	2.68 (0.58)
Training for any staff on non-drug practices to address agitation/behaviors	8.68 (0.82)	94.7	2.68 (0.48)
**Health care supervisor training and knowledge**	**8.49 (0.78)**	**97.4**	**2.74 (0.45)**
Has had training about non-drug treatments for behaviors	8.53 (0.70)	100.0	2.74 (0.45)
Extent of knowledge regarding non-drug treatments for behaviors	8.63 (0.60)	100.0	2.74 (0.45)
Has had training about the side effects of drug treatment for behaviors	8.37 (1.07)	94.7	2.74 (0.45)
Extent of knowledge regarding drug treatment side effects for behaviors	8.42 (1.02)	94.7	2.74 (0.45)
**Recording of health information in chart**	**8.43 (0.74)**	**94.7**	**2.86 (0.29)**
Weight recorded in chart	8.00 (1.56)	89.5	2.94 (0.24)
Vital signs recorded in chart	8.11 (1.20)	89.5	2.89 (0.32)
Emergency department visits recorded in chart	8.63 (0.96)	94.7	2.89 (0.32)
Hospitalizations recorded in chart	8.84 (0.37)	100.0	2.83 (0.38)
Falls recorded in chart	8.74 (0.56)	100.0	2.88 (0.33)
Telephone contact with medical providers recorded in chart	8.37 (1.12)	94.7	2.83 (0.38)
Behavior of residents receiving antipsychotics recorded in chart	8.32 (1.06)	94.7	2.78 (0.43)
**Informs a responsible party when there are changes to status**	**8.16 (1.20)**	**91.2**	**2.84 (0.34)**
Informs a responsible party when there are changes to cognition, behavior, or mood	8.21 (1.13)	94.7	2.78 (0.43)
Informs a responsible party when a fall occurs	8.00 (1.41)	84.2	2.89 (0.32)
Informs a responsible party when there are other changes to medical status	8.26 (1.66)	94.7	2.88 (0.33)
**Other formal assessment tools (other than for cognition)**	**7.63 (1.40)**	**81.1**	**2.74 (0.38)**
Uses formal assessment tool for depression	7.58 (2.19)	78.9	2.68 (0.48)
Uses formal assessment tool for pressure ulcer risk	7.61 (1.38)	77.8	2.68 (0.48)
Uses formal assessment tool for falls risk	7.94 (1.92)	88.9	2.84 (0.37)
Uses formal assessment tool for presence of advance directives	7.50 (2.38)	83.3	2.74 (0.45)
Uses formal assessment tool for elopement risk	7.22 (2.05)	72.2	2.74 (0.45)

Notes: Aggregate items are ordered based on importance rating; individual items are listed in order as presented to participants. SD=standard deviation. A total of 18 of 22 items (81.8%) were rated by all 19 respondents; for other items, the number of respondents was 18. Importance reflects the extent to which the item is expected to significantly affect quality of care outcomes if implemented, considering the extent of expected need and the expected magnitude of benefit, scored 1-9, with 1 being least important and 9 being most important. Feasibility reflects the extent to which the item is feasible for (can potentially be implemented in) no assisted living (AL) communities, some AL communities, or all AL communities, based on factors such as variable case mix, location, or other considerations, scored as “none” (1), “some” (2), and “all” (3) communities.

© 2022 Zimmerman S et al. *JAMA Network Open.*

eTable 4. Items Rated of Medium Importance (≥ 4.0, < 7.0), by Domain (N=19)

Domains and Items (listed in order as presented to participants)	Importance (rated 1-9) Mean (SD)	Feasibility (rated 1-3) Mean (SD)
**Community Demographics & Administration**		
Assisted living community has memory care unit/designated dementia beds	5.95 (1.87)	2.11 (0.32)
Number of years as administrator at that assisted living community	5.79 (2.23)	2.39 (0.50)
Administrator is a licensed nursing home administrator	4.26 (2.84)	2.28 (0.46)
Administrator is a LPN/LVN	4.37 (2.22)	2.18 (0.39)
Administrator is a RN	4.95 (2.72)	2.00 (0.00)
Administrator has a Bachelor of Science in Nursing	4.63 (2.65)	2.00 (0.00)
Administrator has a Master of Science in Nursing	4.21 (2.86)	2.00 (0.00)
Administrator is an Advanced Practice Nurse	4.11 (3.03)	1.89 (0.32)
Administrator is a Physician Assistant	4.21 (2.90)	1.89 (0.32)
Number of years as health care supervisor at that assisted living community	6.32 (2.19)	2.24 (0.44)
Health care supervisor is a LPN/LVN	6.37 (2.22)	2.33 (0.49)
Health care supervisor is a RN	6.37 (2.31)	2.11 (0.32)
Health care supervisor has a Bachelor of Science in Nursing	5.74 (2.45)	2.06 (0.24)
Health care supervisor has a Master of Science in Nursing	5.21 (2.84)	1.89 (0.32)
Health care supervisor is an Advanced Practice Nurse	5.58 (3.15)	1.78 (0.43)
Health care supervisor is a Physician Assistant	5.11 (3.11)	1.83 (0.38)
**Staffing & Staff Training**		
If RN on-site, has RN available part-time	6.42 (2.24)	2.16 (0.37)
If LPN/LVN on-site, LPN/LVN available part-time	6.47 (2.04)	2.32 (0.48)
If LPN/LVN on-site, LPN/LVN on-call (if on-site LPN/LVN is not present)	5.72 (2.27)	2.47 (0.51)
Percent of direct care workers who are contract staff[a]	5.68 (2.96)	2.26 (0.56)
Percent of direct care workers who are medication technicians[a]	6.16 (2.27)	2.39 (0.50)
Training on side effects of drug treatment for staff who do not administer medications	6.47 (1.61)	2.58 (0.51)
**Nursing & Related Services**		
Mobile X-ray comes on-site	5.89 (2.47)	2.21 (0.42)
Catheter insertion to obtain urine specimen on-site	6.11 (2.62)	2.05 (0.40)
Provision of intramuscular injections on-site	6.16 (2.52)	2.26 (0.56)
Administration of intravenous fluids on-site	5.11 (2.21)	2.05 (0.40)
Administration of tube feedings on-site	5.42 (2.43)	1.95 (0.23)
Provision of care for stage 2/3 pressure ulcers on-site	6.58 (2.24)	2.16 (0.37)
If schedule off-site visits, assisted living provides transportation for off-site visits	6.68 (2.33)	2.11 (0.32)
If schedule off-site visits, assisted living staff accompany residents for off-site visits	6.95 (1.58)	1.95 (0.23)
**Resident Assessment & Care Planning**		
Conducts quarterly formal resident assessment	6.42 (2.34)	2.53 (0.61)
Conducts twice a year formal resident assessment	6.32 (2.50)	2.68 (0.48)
Conducts annual formal resident assessment	6.11 (3.02)	2.84 (0.37)
Conducts quarterly formal resident care or service plan meeting[a]	6.79 (2.25)	2.63 (0.50)
Conducts twice a year formal resident care or service plan meeting[a]	6.37 (2.39)	2.68 (0.48)
Conducts annual formal resident care or service plan meeting[a]	6.58 (3.01)	2.89 (0.32)
PCP (if medical needs) present during assessment/care planning	6.11 (2.35)	1.95 (0.62)
Mental health provider (if related needs) present during assessment/care planning	6.42 (2.41)	2.05 (0.52)
Rehabilitation provider (if related needs) present during assessment/care planning	6.47 (2.09)	2.11 (0.46)
**Policies & Practices**		
Has an antibiotic stewardship program in place	6.21 (2.68)	2.33 (0.59)
Frequency with which pharmacist conducts formal med review = 1-3 times a year	6.22 (2.26)	2.29 (0.69)
Frequency with which pharmacist conducts formal med review = 12+ times a year	4.78 (2.46)	1.71 (0.47)
Requires informed consent from residents before prescribing a new medication	5.68 (3.32)	2.29 (0.77)
Requires informed consent from residents for new psychoactive medications		
Requires informed consent from residents for new antipsychotic medications[a]	6.58 (3.08)	2.50 (0.51)
Requires informed consent from residents for new opioid medications[a]	6.58 (3.11)	2.53 (0.51)
Requires all residents to have advance directives	6.68 (3.09)	2.38 (0.72)
Has a policy/procedure regarding marijuana use	6.95 (2.55)	2.58 (0.51)
Policy/procedure to have PRN medications available for behavior		
Has policy/procedure requiring residents to have PRN available for agitation[a]	5.32 (3.45)	2.44 (0.70)
Has policy/procedure requiring residents to have PRN available for insomnia[a]	5.05 (3.14)	2.33 (0.77)
Informs a responsible party when on-site health care provider visits	6.58 (2.48)	2.41 (0.51)
Requires informed consent from families before prescribing a new medication	5.00 (3.43)	2.19 (0.66)
Requires informed consent from families for new psychoactive medications		

© 2022 Zimmerman S et al. *JAMA Network Open.*

Domains and Items (listed in order as presented to participants)	Importance (rated 1-9) Mean (SD)	Feasibility (rated 1-3) Mean (SD)
Requires informed consent from families for new antipsychotic medications[a]	5.89 (3.31)	2.44 (0.63)
Requires informed consent from families for new opioid medications[a]	5.95 (3.27)	2.44 (0.63)
RN most commonly documents residents' care	5.79 (2.95)	2.00 (0.34)
LPN/LVN most commonly documents residents' care	6.26 (2.73)	2.28 (0.46)
Person who documents resident's care		
Medication technician most commonly documents residents' care	5.21 (2.42)	2.22 (0.43)
Certified nursing assistant most commonly documents residents' care	5.05 (2.59)	2.28 (0.57)
Personal care aide most commonly documents residents' care	5.47 (2.37)	2.35 (0.49)
Food consumption recorded in chart	6.37 (2.48)	2.56 (0.62)
Uses electronic health records for medication prescribing and administration	5.84 (2.67)	2.00 (0.00)
Uses electronic health records for resident care notes written by assisted living staff	5.21 (2.32)	2.00 (0.00)
Uses electronic health records for resident care notes written by health care provider	6.00 (2.30)	2.00 (0.00)
**Medical and Mental Health Providers & Care**		
Residents receive care from same PCP as prior to admit	5.68 (2.11)	2.06 (0.24)
Resident-to-unique PCP ratio[a]	4.26 (2.68)	1.94 (0.24)
Has PCPs who treat only long-term care residents	5.32 (2.65)	2.00 (0.00)
MD/DO treats majority of residents	5.37 (2.59)	2.06 (0.24)
NP or PA treatment		
NP treats majority of residents	5.53 (2.06)	2.00 (0.00)
PA treats majority of residents	4.84 (1.89)	1.94 (0.24)
Has a medical director or equivalent[a]	6.00 (3.04)	2.11 (0.32)
If medical director, is an MD	6.16 (3.00)	2.11 (0.32)
If medical director, is on-site at least weekly	5.89 (3.03)	2.00 (0.35)
If medical director, is on-site at least monthly	6.37 (2.50)	2.11 (0.32)
If medical director, practice only serves long-term care residents	5.84 (3.10)	2.06 (0.24)
Percent of PCPs who come on-site[a]	6.47 (2.67)	2.12 (0.33)
PCP is on-site at least weekly[a]	6.21 (2.95)	1.94 (0.24)
PCP is on-site at least monthly[a]	6.89 (2.18)	2.17 (0.38)
If medical care is provided on-site, it is provided at least once/week	6.18 (2.53)	2.06 (0.43)
PCP works in a practice that treats only long- term care residents	6.05 (2.57)	2.00 (0.00)
Percent of residents receiving on-site care[a]	5.74 (2.81)	2.11 (0.32)
Has a special room dedicated for care	5.00 (2.47)	2.00 (0.00)
Medical providers provide in-service training to staff	6.84 (2.52)	2.11 (0.32)
Residents receive mental health care from same provider as prior to admit	6.63 (1.71)	2.06 (0.24)
Residents requiring mental health care-to-unique mental health provider ratio[a]	5.00 (2.43)	1.94 (0.43)
Has mental health providers who treat only long-term care residents	6.05 (2.53)	1.94 (0.42)
Psychiatrist treats majority of residents	4.42 (2.89)	1.94 (0.24)
Psychologist treats majority of residents	5.26 (2.38)	2.06 (0.24)
Social worker treats majority of residents	5.47 (2.12)	2.06 (0.24)
Counselor treats majority of residents	4.68 (2.36)	2.06 (0.24)
Percent of mental health providers who come on-site[a]	6.11 (2.66)	2.00 (0.34)
A mental health provider is on-site at least weekly[a]	5.84 (2.83)	2.06 (0.42)
A mental health provider is on-site at least monthly[a]	6.16 (2.59)	2.24 (0.44)
If mental health care is on-site, it is provided at least once/week	6.06 (2.44)	2.06 (0.43)
Limited number of consistent mental health providers treat majority	6.74 (1.82)	2.05 (0.23)
A mental health provider works in a practice that treats only long-term care residents	5.21 (3.05)	1.94 (0.24)
Percent of residents needing mental health care who receive on-site care[a]	6.47 (2.55)	2.17 (0.38)
Mental health providers provide in-service training to staff	6.84 (2.43)	2.18 (0.39)
Mental health providers participate in quality improvement efforts	6.84 (2.27)	2.17 (0.38)
Uses telemedicine with medical or mental health providers	6.84 (2.43)	2.28 (0.46)
Uses fax to communicate with medical/mental health providers	5.21 (2.94)	2.47 (0.51)
Uses text message to communicate with medical/mental health providers	5.00 (2.87)	2.18 (0.39)
Uses e-mail to communicate with medical/mental health providers	6.32 (2.54)	2.31 (0.48)

Notes: DO=Doctor of Osteopathic Medicine; LPN=licensed practical nurse; LVN=licensed vocational nurse; MD=Doctor of Medicine; NP=nurse practitioner; PA=physician assistant; PCP=primary care provider; RN=registered nurse; SD=standard deviation. Excepting the two indented items, 102 of 116 items (87.9%) were rated by all 19 respondents; for other items, the number of respondents ranged from 17-18. Importance reflects the extent to which the item is expected to significantly affect quality of care outcomes if implemented, considering the extent of expected need and the expected magnitude of benefit, scored 1-9, with 1 being least important and 9 being most important. Feasibility reflects the extent to which the item is feasible for (can potentially be implemented in) no assisted living (AL) communities, some AL communities, or all AL communities, based on factors such as variable case mix, location, or other considerations, scored as "none" (1), "some" (2), and "all" (3) communities.

[a] Further explored in Round 2; for two of the items, the revised question and responses are indented (N=17).

© 2022 Zimmerman S et al. *JAMA Network Open.*

eTable 5. Items Rated of Low Importance (< 4.0), by Domain (N=19)

Domains and Items (listed in order as presented to participants)	Importance (rated 1-9) Mean (SD)	Feasibility (rated 1-3) Mean (SD)
**Community Demographics & Administration**		
Assisted living community is affiliated with a nursing home	3.05 (2.27)	2.00 (0.00)
Assisted living community is affiliated with a hospital	2.89 (2.16)	1.94 (0.24)
Assisted living or parent organization operates a home care agency	3.11 (2.00)	2.00 (0.00)
Health care supervisor is a licensed nursing home administrator	3.05 (1.96)	2.00 (0.50)
**Policies & Practices**		
Frequency with which pharmacist conducts formal med review = Never	2.44 (2.59)	2.18 (0.81)
Has a policy/procedure requiring a visit to an emergency department after a fall	3.95 (2.68)	2.22 (0.65)
Standard policies are only for residents who are not cognitively intact	3.19 (3.04)	2.07 (0.96)

Notes: SD=standard deviation. A total of 5 of 9 items (55.6%) were rated by all 19 respondents; for other items, the number of respondents ranged from 16-18. Importance reflects the extent to which the item is expected to significantly affect quality of care outcomes if implemented, considering the extent of expected need and the expected magnitude of benefit, scored 1-9, with 1 being least important and 9 being most important. Feasibility reflects the extent to which the item is feasible for (can potentially be implemented in) no assisted living (AL) communities, some AL communities, or all AL communities, based on factors such as variable case mix, location, or other considerations, scored as “none” (1), “some” (2), and “all” (3) communities.

© 2022 Zimmerman S et al. *JAMA Network Open.*
